# Meta-analysis of programmed cell death 1 polymorphisms with systemic lupus erythematosus risk

**DOI:** 10.18632/oncotarget.16378

**Published:** 2017-03-18

**Authors:** Jie Gao, Nan Gai, Li Wang, Kang Liu, Xing-Han Liu, Lin-Ting Wei, Tian Tian, Shan-Li Li, Yi Zheng, Yu-Jiao Deng, Zhi-Jun Dai, Rong-Guo Fu

**Affiliations:** ^1^ Department of Nephrology, Second Affiliated Hospital of Xi'an Jiaotong University, Xi'an, China; ^2^ Department of Rheumatic Immunology, Xi'an No.5 Hospital, Xi'an, China; ^3^ Department of Oncology, Second Affiliated Hospital of Xi'an Jiaotong University, Xi'an, China

**Keywords:** PDCD1, SLE, polymorphism, risk, meta-analysis

## Abstract

The association of polymorphisms in programmed cell death 1 (PDCD1) gene with systemic lupus erythematosus (SLE) risk is inconsistent across different studies. This meta-analysis is aimed to provide reliable evidence to the association of five common PDCD1 polymorphisms (PD1.1, PD1.2, PD1.3, PD1.5 and PD1.6) with SLE risk. A total of 28 studies with 4,344 SLE cases and 5,474 healthy controls were included in this meta-analysis. PD1.3 polymorphism was significantly associated with SLE in the overall population (A *vs*. G: OR = 1.35, 95% CI = 1.12-1.63; GA *vs*.GG: OR = 1.41, 95% CI = 1.12-1.76; AA+GA *vs*. GG: OR = 1.41, 95% CI = 1.13-1.7). In the stratified analyses based on ethnicity, we found a significant association in Caucasians and in Mexicans. In the subgroup analyses by gender, a significant association was found between PD1.3 polymorphism and SLE risk in males. The results also suggested an association between the PD1.6 polymorphism and decreased SLE risk (A *vs*. G: OR = 0.84, 95% CI = 0.73-0.96). Our meta-analysis revealed that PD1.3 polymorphism may increase the susceptibility to SLE, particularly in Caucasians, while PD1.6 may be a protective factor to SLE.

## INTRODUCTION

Systemic lupus erythematosus (SLE) is a multisystem autoimmune disease. And its characteristics are autoantibody production, hyperactive T and B cells, immune complex deposition and multi-organ damage (lupus nephritis, coronary artery disease and osteoporosis) [1]. SLE is the prototype of autoimmune diseases, characterized by the presence of a wide range of clinical manifestations. Women of reproductive age are mostly affected by SLE, and up to 20% of the cases begin in childhood [2]. About 50% of SLE patients will develop into the most severe symptoms of the disease, such as nephritis and cerebral vasculitis [3]. Multiple factors, including genetic, hormonal and environmental factors can influence the pathogenesis of SLE [4]. Although the detailed etiology is still obscure, many genes are related to the etiopathogenesis of this disease.

Programmed cell death 1 (PDCD1), which is also called PD-1, is located on the chromosome 2q37 region. And it is recently suggested to be an important candidate for SLE susceptibility. *PDCD1* encodes PD-1 molecule, which has a tyrosine-based inhibitory motif, is an immunoinhibitory receptor belonging to the CD28/B7 family. *PDCD-1* is inductively expressed on CD4^+^/ CD8^+^ T cells, NKT cells, B cells and monocytes after being activated [5]. It plays a critical role in regulating peripheral self-tolerance in T and B cells through activation-induced cell death or allergy in autoimmunity prevention [6–8]. And mice that are lack of *PDCD1* can develop an SLE-like disease [9].

The PD-1/PD-L pathway comprises PD-1 receptor and two ligands of it: PD-L1 and PD-L2. PD-1 receptor is inductively expressed on the activated T- and B cells, as well as myeloid cells. *PDCD-1* is widely expressed on T cells in comparison to the specific expression of other members in CD28 family. And its expression is much broader than PD-L2 in PD-Ls. Both PD-L1 and PD-1 are expressed on CD4+CD25+ T cells, but it is not clear whether they can influence the function of these regulatory T cells [5].

There are several common polymorphisms in *PDCD-1*: PD-1.1 is located in the promoter (position -531 from the translation start), PD-1.2 is located in intron 2 (position 6,438), PD-1.3 is located in intron 4 (position 7,146), PD-1.4 is located in intron 4 (position 7,499), PD-1.5 is located in exon 5 (position 7,785, alanine→alanine) and PD-1.6 is located in the 3′UTR (position 8,738) [10]. Recently, a number of case-control studies have investigated the relationship of *PDCD1* polymorphisms with the susceptibility of SLE, but the results were inconsistent [10–14]. Prokunina *et al*. [10]first identified an allelic variant PD1.3 and found that this polymorphism was related to the SLE development in Europeans and Mexicans. Vela’zquez-Cruz's *et al*. [11]findings also support an association between the PD1.3 polymorphism and the susceptibility to childhood-onset SLE in Mexicans. However, Mostowska *et al*. [12] did not observe any correlation between SLE risk and PD1.3 polymorphism.Chua *et al*. [13] supported that PD1.5 variant may be a risk factor to SLE susceptibility, and the PD1.1, PD1.3, and PD1.6 did not associate with SLE risk in Malaysian cohort. But Mahmoudi*et al*. [14] found that PD1.1 polymorphism played a key role in the juvenile-onset SLE development in Iranian population. Based on these findings, the effects of *PDCD1* polymorphisms have been widely discussed, but no conclusive relationships have been determined. In addition, the sample size in most of these studies were relatively small, thus were not powerful enough to assess whether there is an association between SLE and *PDCD1* polymorphisms.

Herein, the aim of this meta-analysis is to provide a reliable estimation of the association of five common *PDCD1* polymorphisms (PD1.1, PD1.2, PD1.3, PD1.5 and PD1.6) with SLE risk by combining original data from relevant primary studies and achieve a more comprehensive evaluation.

## RESULTS

### Characteristics of the included studies

**Figure 1:**
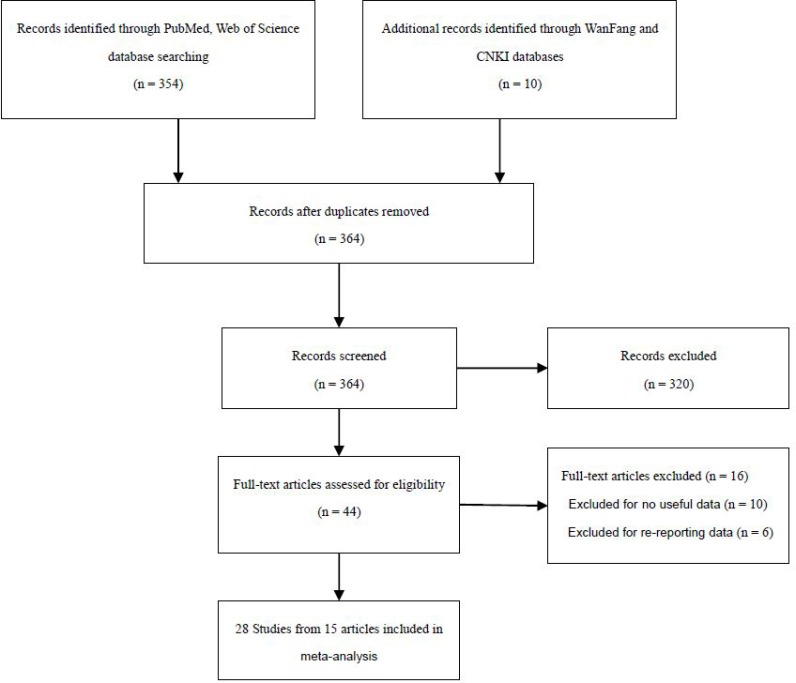
Preferred Reporting Items for Systematic Reviews and Meta-Analyses flow diagram of the literature review process for PDCD1 polymorphism and systemic lupus erythematosus CNKI = China National Knowledge Infrastructure.

All studies in this meta-analysis were published prior to March 30, 2016 through a systematic search in the PubMed, Web of Science, WanFang and CNKI databases. 364 articles were identified that evaluated the association of *PDCD1* polymorphisms and SLE. The study selection process was shown in Figure 1. According to the Preferred Reporting Items for Systematic Reviews and Meta-analyses (PRISMA) guidelines, after manually screening the titles and abstracts, 320 studies were ultimately excluded. After reading the full texts of the remaining 44 articles, 16 were excluded due to lack of complete necessary data (10 articles) or because of re-reporting data (6 articles). Finally, as shown in Table 1, a total of 28 studies [7, 10–23]with 4,344 cases and 5,474 heathy controls were found to meet the inclusion criteria for assessing the influence of the five *PDCD1* polymorphisms on SLE risk.

**Table 1:** Characteristics of the studies included in the meta-analysis

First author	Year	Country	Ethnicity	Genotyping medthod	Source of control	Total sample size (case/control)	SNP No.
Prokunina-1[10]	2002	Norway	Caucasian	PCR-RFLP	Population	32/32	3,4,5
Prokunina-2	2002	Sweden	Caucasian	PCR-RFLP	Population	263/235	3,4,5
Prokunina-3	2002	America	Caucasian	PCR-RFLP	Population	145/80	3,4,5
Prokunina-4	2002	Mexico	Mixed	PCR-RFLP	Population	402/149	3,4,5
Prokunina-5	2002	America	African	PCR-RFLP	Population	80/18	3,4,5
Ferreiros-Vidal[7]	2004	Spain	Caucasian	PCR-RFLP	Population	518/800	3
Nielsen [23]	2004	Denmark	Caucasian	sequencing	Population	95/155	3,4,5
Sanghera-1 [22]	2004	America	Caucasian	PCR-RFLP	Population	276/359	3
Sanghera-2	2004	America	African	PCR-RFLP	Population	35/31	3
Johansson [24]	2005	Sweden	Caucasian	TaqMan	Population	260/670	3
Wang20	2006	Taiwan	Asian	PCR-RFLP	Hospital	109/100	1,2,3,4,5
Ferreiros-Vidal-1 [21]	2007	Germany	Caucasian	PCR-RFLP	Population	101/100	3
Ferreiros-Vidal-2	2007	Czech R	Caucasian	PCR-RFLP	Population	101/100	3
Ferreiros-Vidal-3	2007	Greece	Caucasian	PCR-RFLP	Population	198/380	3
Ferreiros-Vidal-4	2007	Hungary	Caucasian	PCR-RFLP	Population	93/99	3
Ferreiros-Vidal-5	2007	Italy	Caucasian	PCR-RFLP	Population	86/218	3
Ferreiros-Vidal-6	2007	Italy	Caucasian	PCR-RFLP	Population	128/211	3
Ferreiros-Vidal-7	2007	Italy	Caucasian	PCR-RFLP	Population	76/102	3
Velazquez-Cruz [11]	2007	Mexico	Mixed	TaqMan	Population	250/355	3,4,5
Kong [18]	2008	China	Asian	PCR-RFLP	Hospital	164/163	2
Kong [19]	2008	China	Asian	PCR-RFLP	Hospital	132/160	4,5
Mostowska [12]	2008	Poland	Caucasian	PCR-RFLP	Population	102/140	2,3,5
Bertsias [17]	2009	Greece	Caucasian	PCR-RFLP	Population	289/256	3
Peng [16]	2010	China	Asian	PCR-RFLP	Hospital	159/159	2,4,5
Chua-1[13]	2015	China	Asian	TaqMan	Population	115/115	1,3,4,5
Chua-2	2015	Malaysia	Asian	TaqMan	Population	70/70	1,3,4,5
Chua-3	2015	India	Asian	TaqMan	Population	15/15	1,3,4,5
Mahmoudi [14]	2015	Iran	Asian	PCR-RFLP	Population	50/202	1,3

CC: case-control, PCR: polymerase chain reaction, RFLP: restriction fragment length polymorphism. SNP: single-nucleotide polymorphisms; SNP No.1: rs 36084323 G > A (PD1.1); 2: rs 34819629 G > A (PD1.2); 3: rs 11568821 G>A (PD1.3); 4: rs 2227981 C > T (PD1.5); 5: rs 10204525 G > A (PD1.6)

Among the eligible 28 studies, 16 were carried out in Caucasians. Eight studies were based on subjects with an Asian background, 7 were performed in China and one was from Iran. Only one study came from African American and two from Mexico. The NOS score of all articles was more than 6, indicating each included study was of high-quality. The characteristics of the included studies were shown in Table 1. All of the included eligible studies were published in English or Chinese. All studies were case-control studies. Among these studies, 5 studies were about PD1.1, 4 about PD1.2, 25 about PD1.3, 13 about PD1.5, and 14 about PD1.6. There were 4 hospital-based studies and 24 population-based studies.

### Meta-analysis of the PD1.1, PD1.2 polymorphisms and SLE risk

There were five studies with 359 cases and 502 controls for PD1.1, four studies contained 534 cases and 562 controls for PD1.2. The average minor allele frequency were 0.41 and 0.52, respectively (Table 1). There was no significant association in the two polymorphisms under all allele/genotype comparisons (*P* > 0.05). Moreover, the Hardy-Weinberg equilibrium (HWE) of one study about PD1.2 was 0.01 (*P*_HWE_ < 0.05). We omitted this study and the results were in accordance with the overall population before omitting this study (Table 2).

**Table 2:** Meta-analysis of the association between *PDCD1* polymorphisms and systemic lupus erythematosus risk

Comparisons	OR	95%CI	*P* value	Heterogeneity		Effects model
				*I*^2^	*P* value	
**B vs A**						
PD1.1	1.23	0.82–1.84	0.31	54%	0.07	R
PD1.2	0.80	0.49-1.29	0.36	78%	0.00	R
PD1.3	**1.35**	**1.12-1.63**	0.00	57%	0.00	R
Caucasian	**1.27**	**1.05-1.57**	0.02	62%	0.00	R
Mixed	**2.92**	**1.76-4.86**	0.00	0%	0.74	F
African	2.25	0.64-7.96	0.21	0%	0.92	F
Asian	0.97	0.59-1.62	0.92	0%	0.91	F
PD1.3(gender)	1.36	0.98- 1.87	0.06	71%	0.00	R
Female	1.04	0.78- 1.40	0.78	59%	0.01	R
Male	**2.09**	**1.06- 4.12**	0.03	70%	0.00	R
PD1.5	1.07	0.88-1.30	0.49	62%	0.00	R
Caucasian	0.85	0.69-1.04	0.11	0%	0.84	F
Mixed	1.10	0.82-1.48	0.54	44%	0.18	F
Asian	1.18	0.80-1.75	0.41	76%	0.00	R
PD1.6	**0.84**	**0.73-0.96**	0.01	22%	0.21	F
Caucasian	0.85	0.63-1.13	0.25	14%	0.32	F
Mixed	0.84	0.69-1.02	0.09	0%	0.56	F
Asian	0.84	0.63-1.12	0.23	56%	0.04	R
**BB vs AA**						
PD1.1	1.19	0.75-1.88	0.46	0%	0.77	F
PD1.2	0.80	0.36-1.78	0.59	51%	0.10	F
PD1.3	1.30	0.59- 2.89	0.52	0%	0.62	F
PD1.5	0.87	0.60- 1.27	0.47	0%	0.71	F
PD1.6	0.79	0.54-1.17	0.24	16%	0.30	F
Caucasian	1.20	0.35-4.17	0.77	0%	0.78	F
Asian	0.71	0.36-1.41	0.33	44%	0.11	F
**AB vs AA**						
PD1.1	1.32	0.73-2.38	0.36	53%	0.08	R
PD1.2	1.23	0.78-1.94	0.37	0%	0.85	F
PD1.3	**1.41**	**1.12- 1.76**	0.00	19%	0.26	F
Caucasian	**1.37**	**1.06- 1.76**	0.02	27%	0.24	F
Asian	0.97	0.55- 1.71	0.93	0%	0.89	F
PD1.5	1.27	0.80-2.00	0.31	80%	0.00	R
PD1.6	0.91	0.70-1.19	0.50	0%	0.89	F
Caucasian	1.05	0.63-1.75	0.85	0%	0.97	F
Asian	0.75	0.48-1.17	0.21	0%	0.80	F
**BB+AB vs AA**						
PD1.1	1.36	0.77-2.41	0.30	55%	0.06	R
PD1.2	0.99	0.64-1.53	0.96	0%	0.40	F
PD1.3	1.41	1.13-1.77	0.00	22%	0.23	F
Caucasian	1.38	1.06-1.78	0.02	32%	0.21	F
Asian	0.97	0.56-1.69	0.92	0%	0.90	F
PD1.5	1.24	0.80-1.91	0.34	79%	0.00	R
PD1.6	0.90	0.70-1.15	0.40	0%	0.63	F
Caucasian	1.08	0.66-1.75	0.77	0%	0.96	F
Asian	0.77	0.50-1.20	0.25	4%	0.39	F
BB vs AB+AA						
PD1.1	1.13	0.77-1.65	0.53	0%	0.90	F
PD1.2	0.63	0.33-1.22	0.17	74%	0.01	R
PD1.3	1.26	0.57-2.79	0.57	0%	0.63	F
PD1.5	0.84	0.59- 1.20	0.33	0%	0.57	F
PD1.6	0.80	0.62-1.04	0.10	30%	0.18	F
Caucasian	1.19	0.35-4.09	0.79	0.0%	0.78	F
Asian	0.82	0.56-1.20	0.31	54%	0.05	R

A: the major allele, B: the minor allele, F: fixed effects model, R: random effects model.

### Meta-analysis of the PD1.3 polymorphism and SLE risk

**Figure 2:**
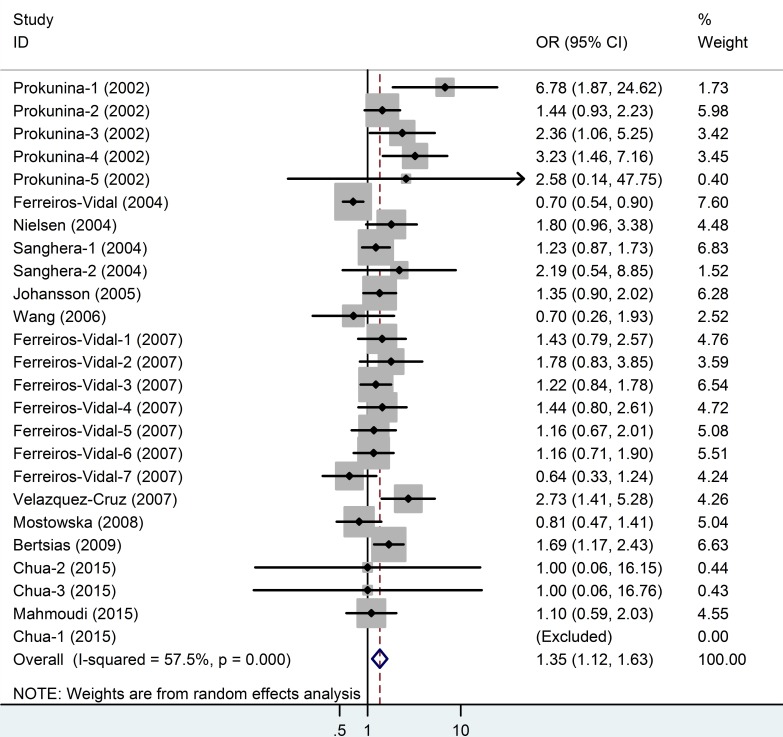
Meta-analysis and pooled relative risk of PD1.3 polymorphism under A *vs* G genetic model CI = confidence interval, OR = odds ratio.

**Figure 3:**
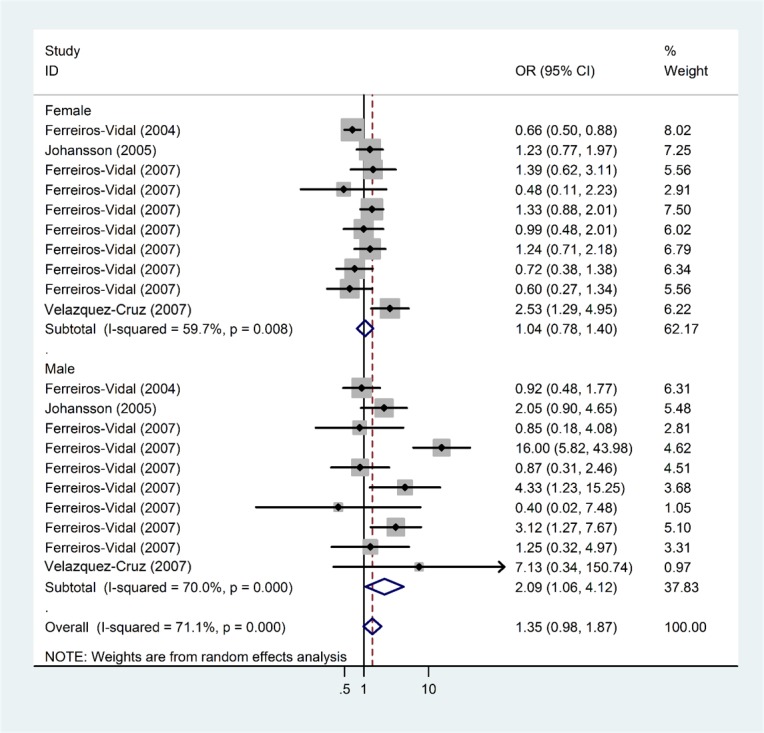
Meta-analysis and pooled relative risk of PD1.3 polymorphism and SLE risk with stratified analysis by gender under A *vs* G genetic model CI = confidence interval, OR = odds ratio.

There were 25 studies with 3,882 cases and 4,968 controls evaluating the effect of *PD*1.3 on SLE risk, and the average minor allele frequency was 0.10(Table 1). As shown in Table 2 and Figure 2, we found a significant association in the overall population (A *vs*. G: OR = 1.35, 95% CI = 1.12-1.63, *P* = 0.00; GA *vs*.GG: OR = 1.41, 95% CI = 1.12-1.76, *P* < 0.001; AA+GA *vs*. GG: OR = 1.41, 95% CI = 1.13-1.77, *P* < 0.001). However, in the stratified analyses based on ethnicity, results demonstrated a significant relationship in Caucasians (A *vs*. G: OR = 1.27, 95% CI = 1.05-1.57, *P* = 0.02; GA *vs*.GG: OR = 1.37, 95% CI = 1.06-1.76, *P* = 0.02; AA+GA *vs*. GG: OR = 1.38, 95% CI = 1.06-1.78, *P* = 0.02) and in Mexicans (A *vs*. G: OR = 2. 92, 95% CI = 1.76-4.86, *P* = 0.00) subgroup, but no association in African America and Asian group. In the subgroup analyses by gender, we found a significant relationship between PD1.3 polymorphism and increased SLE risk in the males (A *vs*. G: OR = 2.09, 95% CI = 1.06-4.12, *P* = 0.03, Figure 3), while there was no association observed in the females (A *vs*. G: OR = 1.04, 95% CI = 0.78-1.40, *P* = 0.78). The distribution of genotypes in the controls conformed to HWE except for one study by Mahmoudi (*P*_HWE_ = 0.02), the results were in accordance with the overall population when we omitted this study (Table 2).

### Meta-analysis of the PD1.5, PD1.6 polymorphism and SLE risk

**Figure 4:**
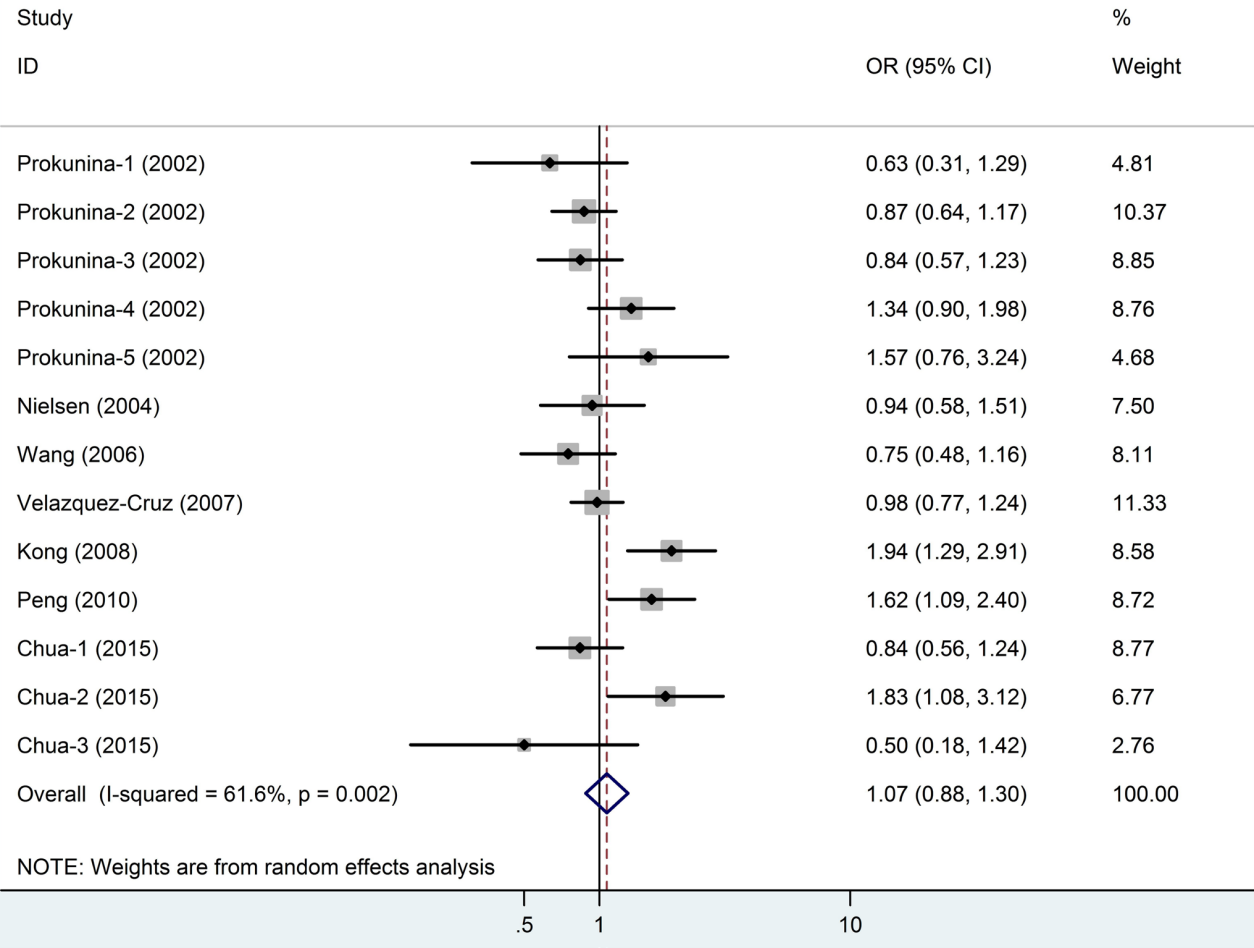
Meta-analysis and pooled relative risk of PD1.5 polymorphism under T *vs* C genetic model CI = confidence interval, OR = odds ratio.

**Figure 5:**
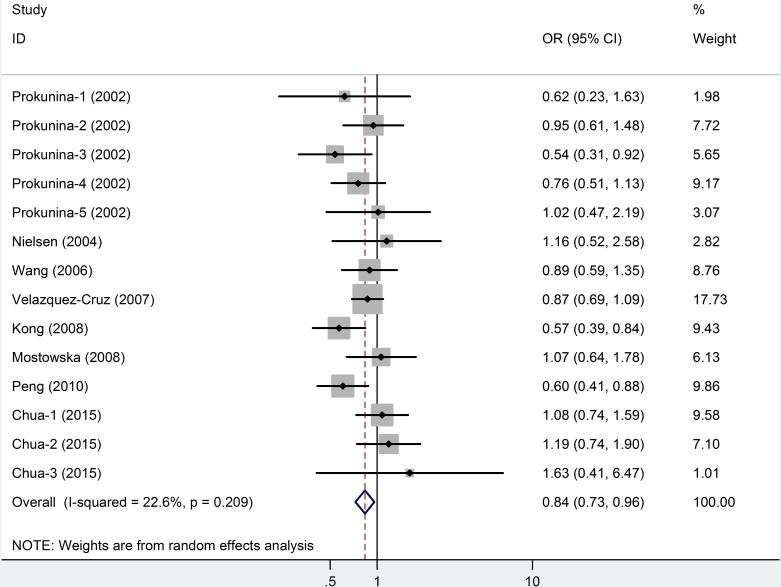
Meta-analysis and pooled relative risk of PD1.6 polymorphism under A *vs* G genetic model CI = confidence interval, OR = odds ratio.

13 studies for PD1.5 contained 1,852 cases and 1,342 controls, and the average minor allele frequency was 0.37. 14 studies for PD1.6 contained 1,923 cases and 1,591 controls, and the average minor allele frequency was 0.41. No significant association was observed under any genotype model in PD1.5 (Figure 4). As shown in Table 2 and Figure 5, an association was observed between the *PD1.6* polymorphism with SLE risk in the overall population (A *vs*. G: OR = 0.84, 95% CI = 0.73-0.96, *P* = 0.01). However, in the subgroup analyses based on ethnicity, we failed to find any significant association in all subgroup.

### Sensitivity analysis

**Figure 6:**
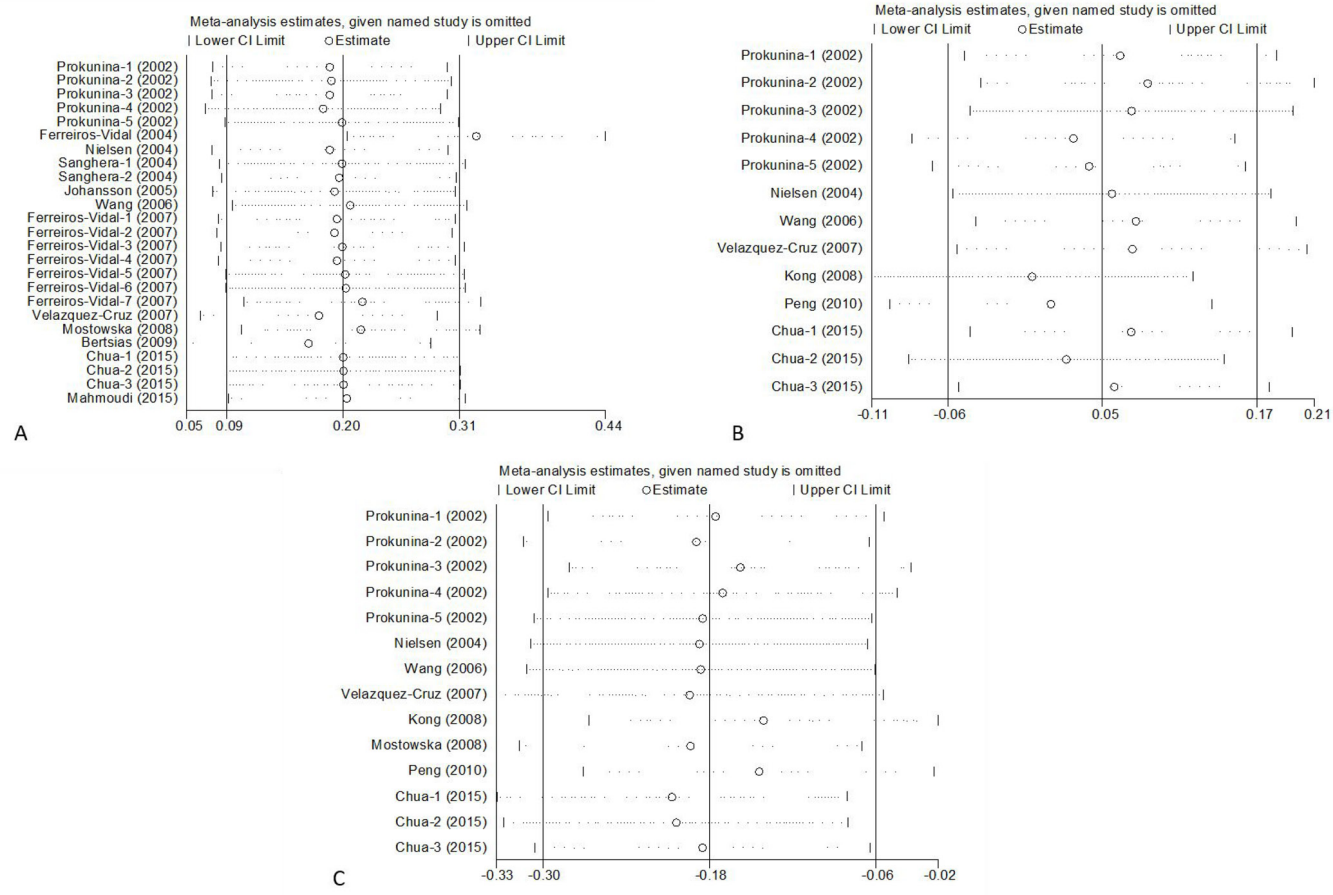
Sensitivity Analysis of *PDCD1* polymorphisms PD1.3 **A.**, PD1.5 **B.**, PD1.6 **C.**

We performed sensitivity analyses by sequentially removing each included study to assess the influence of individual study on the pooled OR for the respective comparisons of the five *PDCD1* polymorphisms. The omission of any study did not have a significant effect on the results, except PD1.3. The results were in accordance with the overall results when Ferreiros-Vidal's study in 2004 (OR = 1.40, 95% CI = 1.20-1.64, *P* = 0.00) was rejected. So the results of this meta-analysis are statistically reliable (Figure 6).

### Heterogeneity analysis and publication bias

The Q test and I^2^ value were used to test the variation in the data caused by heterogeneity. And the results are shown in Table 2. A random-effects model was applied when the *P* value of the test was ≤ 0.1, and the fixed-effects model was used for *P* ≥ 0.1.

**Figure 7:**
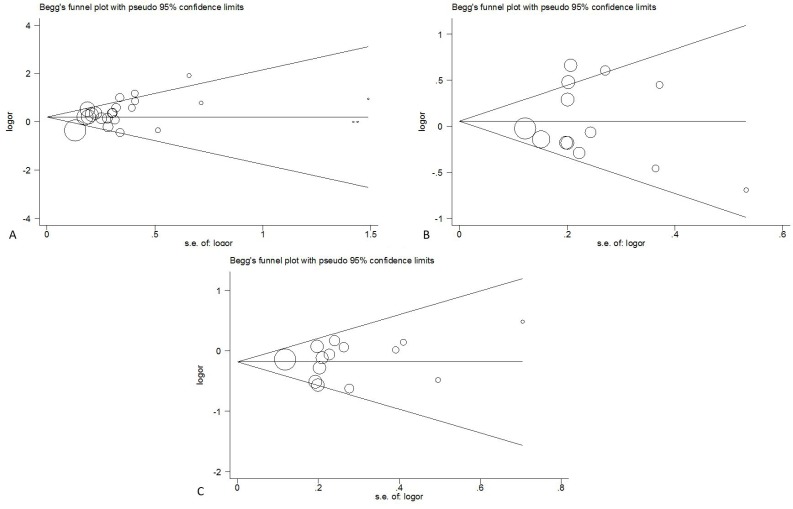
Begg funnel polt for publication bias test of *PDCD1* polymorphisms PD1.3 **A.**, PD1.5 **B.**, PD1.6 **C.**

We constructed a funnel plot and performed Egger's test to evaluate the extent of publication bias in our dataset. As shown in Figure 7, the funnel plots failed to show any obvious asymmetry for the five polymorphisms in the overall population. And the results of Egger's test revealed no publication bias except PD1.3. (*P* = 0.100, 0.451, 0.037, 0.303 and 0.604 for PD1.1, 1.2, 1.3, 1.5 and 1.6).

## DISCUSSION

*PDCD-1* gene, as a new member of the CD28 family, has 23% homologues in amino acid sequence with CTLA-4 [24]. In the cytoplasmic tail of *PDCD-1* gene, there are two characteristic tyrosine domains: one immunotyrosine inhibitory motif and one immune receptor tyrosine-based switch motif [25]. *PDCD-1* gene was isolated from thymus T cell under the stimulation of apoptosis [26]. We considered *PDCD1* to be a strong susceptibility locus for SLE because it belongs to the immunoglobulin family [9]. Mice deficient in *PDCD-1* developed spontaneous autoimmune diseases, which suggested a negative costimulatory function [27]. Disruption of the *PDCD-1* gene in C57BL/6 mice developed lupus-like glomerulonephritis [9]. Furthermore, *PDCD1* act as a crucial regulator of acquired immunity, and its genetic variants may have an effect on other diseases [5].

The *PDCD-1* gene is a SLE susceptible locus in whole genome-wide linkage studies [28]. Several polymorphisms in the *PDCD-1* with SLE has been demonstrated in some studies [29]. PD1.3 polymorphism was reported as a susceptibility factor for SLE in PD1 intron 4, could decrease the expression of *PDCD-1* though affecting RUNX1 binding [30]. Many studies indicated that the *PDCD-1* gene polymorphisms were associated with SLE risk [7, 15–17, 31]. For instance, association between PD1.3 and SLE has recurred in a great deal of previous studies [11, 31]. But other research failed to replicate the results [19, 32]or the association was only with certain SLE subtypes [15–17, 31] in some studies. So these associations have still been considered to be controversial. Therefore we conducted a meta-analysis based on published studies to provide a quantitative assessment on the relationship between *PDCD1* and SLE. As we know, this is the most comprehensive meta-analysis to date to evaluate the association between *PDCD1* polymorphism and SLE risk.

Two previous meta-analyses [33, 34] were conducted almost at the same time(in 2009) to explore the association between *PDCD1* polymorphisms and SLE. Liu et al. [33]paid attention to the effect of PD1.3 polymorphism on SLE risk in European populations by combining results in meta-analysis. They concluded that PD1.3 polymorphism significantly associate with higher SLE risk in non-Spanish European population, while a negative association was observed in Spanish population.In the meta-analysis by Lee et al. [35], they included 15 case-control studies and demonstrated that the PD1.3 and PD1.5 allele was related to SLE susceptibility in Europeans, which was partly consistent with the results of our study.

Our meta-analysis included 28 independent case-control studies involving 4,344 SLE patients and 5,474 healthy controls. We have used Newcastle-Ottawa Scale to assess the quality of our studies and they are all high quality. [36] We analyzed the relationship between *PDCD1* polymorphisms (PD1.1, PD1.2, PD1.3, PD1.5 and PD1.6) and SLE risk. Conversely, PD1.3 polymorphism may increase SLE susceptibility, and PD1.6 polymorphism may be a protective factor against SLE. However, Lee et al. found no relationship between PD1.6 polymorphism and SLE risk in Europeans [35]. This disaccord was probably because that the relatively small sample size may result in insufficient statistical power. So in our study, massive cases and controls were collected from more published case-control studies, which increased statistical power and provided competent evidence for us to obtain a reliable result. In our results, there was no significant correlation between PD1.1, PD1.2, PD1.5 and SLE risk in the overall population.

Overall, there was an increased association between PD1.3 and SLE. In the stratified analyses of race, the association only was found in Caucasians and Mexicans subgroup, and failed in African America and Asian group. Furthermore, of all the eligible studies, only two studies were based on African American population and two studies were based on Mexican. So following we still need more related studies to confirm and supplement this result.

In the subgroup analysis of PD1.3 gender, we found an increased SLE risk in male based on the allele model and no relationship in women. However, as we know, SLE prevalence rate of women is much higher than that of male, it may be the reason that the male subjects was less than the female. Afterwards we may need added the larger sample to evaluated the result. Ethnicity and gender may be the essential biological factor that influences PD1.3 polymorphism through gene-gene interactions, which was particularly prominent in SLE.

The A genotype of PD1.6 was associated with decreased SLE susceptibility (A *vs*. G: OR = 0.84, 95% CI = 0.73-0.96, *P* = 0.01), but no in there Subgroup analyses by ethnicity. Furthermore, in other four genotype models of PD1.6, no significantly increased risk of SLE is associated. There was no marked association between the PD1.1, PD1.2, PD1.5 polymorphism and SLE susceptibility in five genotype models and the overall analysis.

Despite our considerable efforts to search the possible relationship between the *PDCD-1* polymorphisms and SLE risk, some limitations of our work need to be considered. Firstly, potential interactions of *PDCD-1* with other important genes in similar biological process related to SLE risk were not explored because of lacking original data. Due to the pooled data, we are unable to assess the SLE risk by stratification of age, environment factors, SLEDAI score, pathological classification, and other risk factors of SLE. Secondly, the inconsistent designs of the included studies may contribute to the heterogeneity, which might partly reduce the analysis power. Thirdly, publication bias should be concerned. Some unpublished studies and some studies published in other language except English and Chinese were not included in our meta-analysis. Fourthly, the some relative small size (< 200) studies may affect the results. Therefore, further large scale studies from different races, with more available individual data, are needed to validated the association between *PDCD1* polymorphisms and SLE risk.

In conclusion, our meta-analysis provides clear evidence that PD1.3 polymorphism may increase the susceptibility to SLE, particularly in Caucasians, while PD1.6 may be a protective factor to SLE. There was no association between PD1.1, PD1.2, PD1.5 polymorphism with the risk of SLE. Large studies involving more detailed individual data are required to validate our results.

## MATERIALS AND METHODS

### Publication search

A PubMed, Web of Science, Wanfang and Chinese National Knowledge Infrastructure (CNKI) search for studies that examined the association between *PDCD1* gene polymorphisms and SLE risk up to March 30, 2016 was performed using the following search terms: “programmed cell death 1” or “*PDCD1*”, “*PD1*” or “CD279 antigen”, “polymorphism” or “SNPs”, “allele’ or ‘genotype”, “systemic lupus erythematosus” and “SLE”. Only articles published in English or Chinese were eligible for inclusion. Furthermore, the reference lists of all eligible articles, including review articles, were also checked to find additional relevant publications.

### Selection criteria

Studies were considered eligible for further meta-analysis if they met the following criteria: a) studies containing useful genotype frequencies or odds ratio (OR) data; b) case-control studies; c) articles that evaluated the association between *PDCD1* polymorphisms and SLE susceptibility; d) participants in control groups were selected from normal individuals. Non case-control design studies and review papers were excluded. If two or more studies contained overlapping cases or controls, the study with the largest sample size was included in the meta-analysis.

### Data extraction

Two independent investigators (Li Wang and Kang Liu) reviewed the articles, and any discrepant data were discussed by all authors to reach a consensus according to the meta-analysis of observational studies in epidemiology guidelines. [37] The following characteristics were extracted from each included study: first author, year of publication, country of origin, ethnicity, source of controls, number of case and control, and genotypes. Different ethnic groups were categorized as Caucasian, Asian, African, and “mixed”. The Caucasian subgroup included American, Swiss, Spanish, Danish, German, Czech, Greek, Hungarian, Italian (Naples, Milan, Rome), Polish and Nordic. The Asian subgroup included Chinese, Malaysian, Indian, and Iran populations. African American population was classified in the African subgroup and others in Mixed subgroup.

### Data synthesis

Hardy-Weinberg equilibrium (HWE) was firstly assessed in control groups of each study using chi-squared or Fisher's exact test. Based on the genotype and allele frequencies in cases and controls, crude odds ratios (OR) with 95% confidence intervals (95% CI) was used to measure the strength of the associations between *PDCD1* polymorphisms (PD1.1, PD1.2, PD1.3, PD1.5 and PD1.6) and SLE risk. The Z test was used to assess the significance of all pooled ORs and *P* < 0.05 was considered significant.

We assess the association under the allele model (B *vs*. A) among all the studies, with A defined as the major allele and B defined as the minor allele. However, because of the missing information about genotypes, we evaluate the association under other different genetic models (homozygous comparison of BB *vs*. AA, dominant comparison of AB+BB *vs*. AA, recessive comparison of BB *vs*. AA+AB, and heterozygous comparison of AB *vs*. AA) using all the studies except the studies by Prokunina [10] and Ferreiros-Vidal [7].

Statistical heterogeneity among studies was checked using the Q test and I^2^ statistic. When the *P* ≥0.10, a fixed-effect model was used. Otherwise, a random effects model was adopted. In addition, the I^2^-statistic also measures the degree of inconsistency in the studies by computing what percentage of the total variation across studies was due to heterogeneity rather than by chance. A high value of I^2^ indicated a higher probability of the existence of heterogeneity (I^2^ = 0% to 25%, no heterogeneity; I^2^ = 25% to 50%, moderate heterogeneity; I^2^ = 50% to 75%, large heterogeneity; and I^2^ = 75% to 100%, extreme heterogeneity). Potential publication bias was evaluated with Egger's test and the inverted funnel plot, and *P* < 0.05 was considered statistically significant. All statistical analyses were performed by STATA statistical software Version 12.0 (STATA Corp LP, College Station, TX, USA).
